# Cuffless Blood Pressure Measurement Using a Smartphone-Case Based ECG Monitor with Photoplethysmography in Hypertensive Patients

**DOI:** 10.3390/s21103525

**Published:** 2021-05-19

**Authors:** Zhanna Sagirova, Natalia Kuznetsova, Nana Gogiberidze, Daria Gognieva, Aleksandr Suvorov, Petr Chomakhidze, Stefano Omboni, Hugo Saner, Philippe Kopylov

**Affiliations:** 1 Department of Cardiology, Functional and Ultrasound Diagnostics of N.V. Sklifosovsky, Institute for Clinical Medicine, I.M. Sechenov First Moscow State Medical University, 119435 Moscow, Russia; zhanna.s.n@mail.ru (Z.S.); nana10.11@mail.ru (N.G.); stefano.omboni@iitelemed.org (S.O.); 2 Research Center “Digital Biodesign and Personalized Healthcare”, I.M. Sechenov First Moscow State Medical University, 119991 Moscow, Russia; tusia.13@bk.ru (N.K.); dashkagog@mail.ru (D.G.); petr7747@mail.ru (P.C.); fjk@inbox.ru (P.K.); 3 Centre for Analysis of Complex Systems, I.M. Sechenov First Moscow State Medical University, 119991 Moscow, Russia; yourmedstat@gmail.com; 4 Italian Institute of Telemedicine, 21048 Solbiate Arno, Italy; 5 ARTORG Center for Biomedical Engineering Research, University of Bern, 3008 Bern, Switzerland; 6 Institute for Social and Preventive Medicine, University of Bern, 3012 Bern, Switzerland

**Keywords:** photoplethysmography, pulse wave analysis, blood pressure, blood pressure measurement, portable ECG monitor, PPG monitor, smartphone, CardioQVARK, telemedicine

## Abstract

The availability of simple, accurate, and affordable cuffless blood pressure (BP) devices has the potential to greatly increase the compliance with measurement recommendations and the utilization of BP measurements for BP telemonitoring. The aim of this study is to evaluate the correlation between findings from routine BP measurements using a conventional sphygmomanometer with the results from a portable ECG monitor combined with photoplethysmography (PPG) for pulse wave registration in patients with arterial hypertension. Methods: The study included 500 patients aged 32–88 years (mean 64 ± 7.9 years). Mean values from three routine BP measurements by a sphygmomanometer with cuff were selected for comparison; within one minute after the last measurement, an electrocardiogram (ECG) was recorded for 3 min in the standard lead I using a smartphone-case based single-channel ECG monitor (CardioQVARK^®^-limited responsibility company “L-CARD”, Moscow, Russia) simultaneously with a PPG pulse wave recording. Using a combination of the heart signal with the PPG, levels of systolic and diastolic BP were determined based on machine learning using a previously developed and validated algorithm and were compared with sphygmomanometer results. Results: According to the Bland–Altman analysis, SD for systolic BP was 3.63, and bias was 0.32 for systolic BP. SD was 2.95 and bias was 0.61 for diastolic BP. The correlation between the results from the sphygmomanometer and the cuffless method was 0.89 (*p* = 0.001) for systolic and 0.87 (*p* = 0.002) for diastolic BP. Conclusion: Blood pressure measurements on a smartphone-case without a cuff are encouraging. However, further research is needed to improve the accuracy and reliability of clinical use in the majority of patients.

## 1. Introduction

Hypertension is a leading risk factor for cardiovascular morbidity and mortality [1,2]. Accurate assessment of blood pressure (BP) allows timely diagnosis and appropriate treatment of the disease. Invasive assessment of the central arterial blood pressure is the gold standard for evaluation of systolic and diastolic blood pressure. Due to the invasive approach, the risk of complications is significant [3]. Traditionally, a cuff-based measurement is the method of choice for routine application. However, cuff-based measurements are inconvenient and cause discomfort, which leads to decreased compliance of patients with measurement recommendations. Cuffless BP measurement has the potential to overcome some of these problems, to enable more widespread application under various circumstances and therefore also to facilitate BP surveillance by telemedicine. Different devices for cuffless BP measurement are actually explored. Most of the cuffless BP measurement methods are based on photoplethysmography (PPG) and electrocardiogram (ECG). PPG is an optical method based on the determination of changes of blood volume from systole to diastole in arterioles [4]. Several studies investigate pulse transit time (PTT) as the main indicator for cuffless BP measurement [5,6,7]. PTT it is a time from R-wave in electrocardiogram (ECG) to certain point in PPG. Thus, the measurement of blood pressure is based on the determination of the PPT index, which in turn is determined by the ECG and PPG data [8,9,10,11]. The relationship between PTT and BP has been verified in some studies. However, the most PTT-based devices were not comfortable enough and were not suitable for daily use [12,13,14,15]. According to the literature data, there are models for BP assessment based only on features of PPG. However, most researchers proposed experimental models and investigated small group of people [16,17]. So, the development of convenient and portable devices for cuffless BP measurement is relevant.

CardioQVARK^®^ is a smartphone-case based on single-channel ECG monitor combined with a photoplethysmography monitor. The device synchronously records ECG and PPG and is connected to a smartphone application for registration of gender, height, weight, associated risk factors and ICD-10 code. Registered and acquired parameters are transmitted to a server. Algorithms built into the server are programmed to calculate heart rate, determinate heart rhythm (sinus rhythm or atrial fibrillation), PQRST intervals, number of extrasystoles, type of pulse wave, vascular stiffness, oxygen saturation SpO2, and number of breathing cycles per minute and—based on the combination of ECG and PCG results—blood pressure values.

The aim of this study was to compare results from BP measurements with a cuff-based sphygmomanometer with blood pressure results calculated from ECG and PPG registrations by using the CardioQVARK algorithm.

## 2. Materials and Methods

This is a prospective cohort study performed at the I.M. Sechenov First Moscow State Medical University (Sechenov University), Moscow, Russia. The institutional review board approved the study protocol, and all study participants provided written informed consent.

### 2.1. Study Patients

A total of 512 consecutive patients were recruited from an outpatient clinic and entered the screening procedure. The inclusion criteria were: age >18 years, physician-documented history of arterial hypertension, and written informed consent of the patient to participate in the study. Exclusion criteria were: unwillingness to participate in the study, poor quality of ECG and pulse wave recordings, heart rhythm disturbance at the moment of the study, hand tremor, and pacemaker rhythm. Arterial hypertension was defined by systolic blood pressure (SBP) > 140 mm Hg or diastolic blood pressure (DBP) > 90 mm Hg, physician-documented history of hypertension, or by the use of antihypertensive medications [18]. As in a real world setting, the study includes patients with normal blood pressure, with hypertension and with compensated hypertension.

### 2.2. Blood Pressure Measurement and Data Acquisition

At each session, 3 routine cuff BP measurements with 30 s intervals were performed using a sphygmomanometer with a properly sized cuff and the mean value was selected for comparison. The reference blood pressure was measured using a clinically validated oscillometric BP device (Microlife BP AG1-10) that was worn on the upper arm according to the World Health Organization recommendations. The patient was sitting quietly for 15 min before the measurement. The cuff was placed on the left upper arm, 2 cm above the elbow [19].

**Figure 1:**
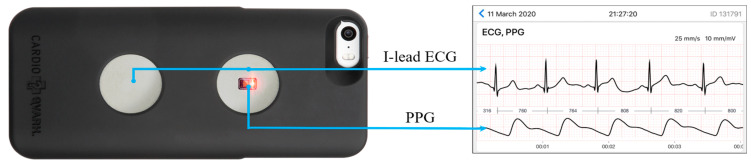
Characteristics of CardioQVARK device. On the **left side**, there is the electrode for I-lead ECG registration and on the **right side** the monitor for photoplethysmography PPG. The device is shown together with an example of the presentation of the recording.

Within one minute after the cuff-based measurements, an ECG in the I standard lead and a photoplethysmocardiogram were recorded simultaneously over 3 min when averaging complexes with CardioQVARK^®^ (Figure 1).

**Figure 2:**
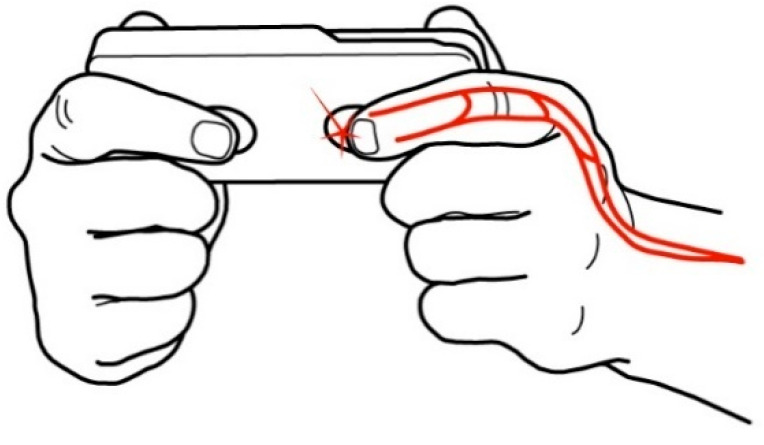
Recording of the ECG and the photoplethysmography image using the CardioQVARK^®^ smartphone case. The index fingers are placed at the ECG electrode (left) and at the photoplethysmograph monitor (right).

ECG signals are recorded from the fingers using one standard ECG lead. The sensors provide a continuous recording of the PPG image of the pulse wave, synchronized with ECG cycles. The setting of recording ECG and PPG is shown in Figure 2.

### 2.3. Characteristics of CardioQVARK Device

The analog-type of the electrocardiography section consists of a low-noise preamplifier and a differential amplifier as a converter driver. The amplification factor of the analog section is 3. Using a 24-bit analog-to-digital converter, a resolution of 0.5623 nV is achieved. The discrimination frequency of the analog-to-digital converter is 1000 Hz, the input impedance is more than 6.5 MOm, the amplitude–frequency characteristic of the analog section is 0.67–320 Hz. The photoplethysmogram is recorded simultaneous with the ECG using the MAX30102 reflective sensor. The wavelength is 880 nm, the bit depth is 16 bits, the sampling rate is 1000 Hz, and the bandwidth is 0–500 Hz.

There are known algorithms for determining blood pressure based on PPT [20,21,22,23,24,25,26,27,28]. There are also patents. One example is the determination of BP by a wearable device, which is a wrist bracelet [24]. The method includes analyzing ECG and PPG to determine a pulse transit time (PTT), a pulse rate (PR), and a diameter parameter.

The algorithm we used in the study is based on simultaneous evaluation of ECG and PPT parameters, which have been recorded with a smartphone case. Device and application are combined to one unit and were registered with the Federal Service for Surveillance in Healthcare № RZN 2019/8124 on 15 February 2019. With the simultaneous analysis of the frequency and time characteristics of the ECG and PW, it became possible to determine the blood pressure by a unique mathematical algorithm, patented by CardioQVARK.

**Figure 3:**
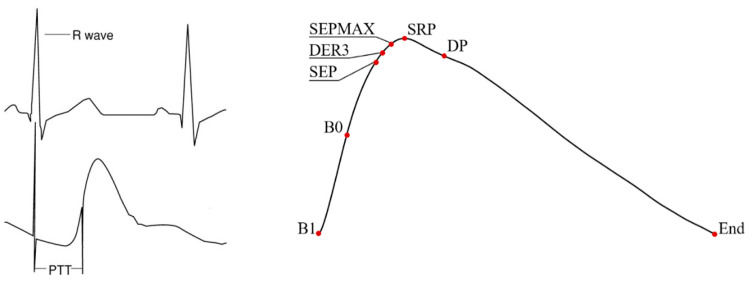
Pulse transit time and pulse wave acquired by photoplethysmography with points of interest. B1 = beginning of the pulse wave, B0 = point of maximum increase of the anterior leg, SEP = peak of the ejection pulse wave, DER3 = first positive peak of the 3th derivate, SEPMAX = first inflection of the pulse wave, SRP = peak of the reflected systolic wave, DP = peak of the diastolic wave, End = end of the pulse wave.

After registration, all ECG and PPG registrations are sent to the server. Each cardiocycle and pulse wave is automatically compared with a standard for data quality. If quality was not sufficient, the respective cardiocycles and/or PPG recordings were deleted. Thereafter, the marking and calculation of the parameters is carried out, on the basis of which value of blood pressure (both systolic and diastolic) is estimated. The figure shows the points of the position of the R-peaks from the ECG and the points of the pulse wave, which we use for analysis. (Figure 3). Point “B1” and point “End” are the standard start and end points for the pulse wave. The rest of the points do not have a specific position and depend on the shape of the pulse wave. They are determined automatically by the algorithm. Each pulse wave is decomposed into elementary waves: forward and backward.

Our algorithm uses ECG parameters (counter analysis) and the following pulse wave parameters: B1 is the beginning of the wave, B0 is the point of maximum increase of the anterior front, SEP is the peak of the ejected pulse wave, DER3 is the first positive peak of the third derivative, SEPMAX is the point of the first inflection of pulse wave, SRP is the peak of the reflected systolic wave, DP is the peak of the diastolic wave, End is the end of the wave. Perfusion index, augmentation index, SBP, and DBP indices are transferred to the client application.

The calculation algorithm of blood pressure is based on the linear regression classical method.

This model has the following features:

BP = A_0_ + A_1_F_1_ + A_2_F_2_ + A_3_F_3_ + … + A_n_F_n_, (1)

where BP is blood pressure and F, F, F, etc., are one of the following features: the time interval from the R-peak to the onset of the PPG wave, the positions of various points of the PPG wave, the logarithms of the amplitudes at the indicated points, and additional spectral and time parameters. A, A, A, etc., are coefficients, calculated in the process of machine learning of the algorithm with the selection of the most significant signs of the ECG and PPG.

Our choice of the linear regression method for calculating blood pressure is due to the characteristics of the available sample: a limited number of records and the well-known limited accuracy of the cuff blood pressure measurements. With more records available, the model can be replaced by other machine learning methods.

An analysis of deviations between two methods in all the group was performed, in the subgroups of patients with BP less than 110/70 mm Hg, in the subgroup with BP between 110/70 and 140/90 mm Hg, and in the subgroup with BP more than 140/90 mm Hg.

### 2.4. Statistical Analysis

We compared systolic and diastolic BP values obtained from a sphygmomanometer with cuff with the values derived from our smartphone-case based BP device.

Statistical analysis was performed in SPSS v. 23 and in R v.4.0.

For parametric data, the Shapiro–Wilk test was used to access the normality of distribution. Mean value (M), standard deviation (SD), median, interquartile range (Me [25%, 75%]), minimum, and maximum values were calculated. For categorical data, the proportions and absolute values were determined.

Spearman’s correlation was used to access the relationship between variables with the calculation of correlation coefficient, r, and its significance.

The Bland–Altman plot was used to evaluate the agreement between cuff and cuffless measurements of systolic and diastolic blood pressure, where the cuff method was the reference method. The mean difference (error) between the cuffless–cuff measurements, its 95% confidence interval (95% CI), and its equality to zero were assessed using the R t.test function [25].

Comparative analysis of subgroups and assessment of error were carried out using the Mann–Whitney–Wilcoxon rank test.

Sensitivity and specificity analysis of the cuffless blood pressure measurement was carried out according to the DeLong method [26] using the R pROC library [27]. Cuff measurements were used as references.

The authors proposed the zonal error grid method associated with estimating the error costs between measurements of the two methods. Zonal error grid is similar to the Clarke error grid analysis. The graph is a scatterplot of cuff and cuffless measurements with selection of five zones A, B, C, D, and E, depending on the error between the methods. Zone A shows deviations of reference 8.3% and lower. Zone B shows deviations that are not of significant clinical importance, 8.4–16.6%. Zone C shows measurements that may influence doctor’s decision, but are not life-threatening, 16.7–33.3%. Zone D shows errors that could be dangerous for the patient, 33.4–41.6%. Zone E shows extreme errors, more than 41.7%. The boundaries of the zones and deviation percent were determined on the basis of blood pressure indicators of the clinical guidelines of the European Society of Cardiology [18].

In our study, hypertension was considered as a BP > 140/90 mm Hg. Prehypertension was considered as a BP > 130/85 mm Hg.

## 3. Results

The study included 500 patients with arterial hypertension. Twelve initially screened patients had the be excluded for the following reasons: unwillingness to participate in the study (4), poor quality of ECG and pulse wave recordings (4), heart rhythm disturbance at the moment of the study (4), hand tremor (0), and pacemaker (0). The age of the study participants ranged from 32 to 88 years (mean 64 ± 7.9 years), 56% were females. All patients who took part were on antihypertensive drugs including angiotensin converting enzyme inhibitors, angiotensin receptor blockers, calcium antagonists, beta blockers, and/or thiazide diuretics.

The mean SBP in our patients was 125 ± 18.8 mm Hg (range 90–175 mm Hg) and the mean DBP was 76 ± 12.4 mm Hg (range 54–105 mm Hg) by using the cuff-based device. The mean SBP was 122.5 ± 17.8 mm Hg (range 87–169 mm Hg) and the mean DBP was 74 ± 11.5 mm Hg (range 56–99 mm Hg) when measured by the “CardioQVARK” monitor.

Reference values were <110/70 mm Hg. Or >140/90 mm Hg measured by sphygmomanometer.

**Figure 4:**
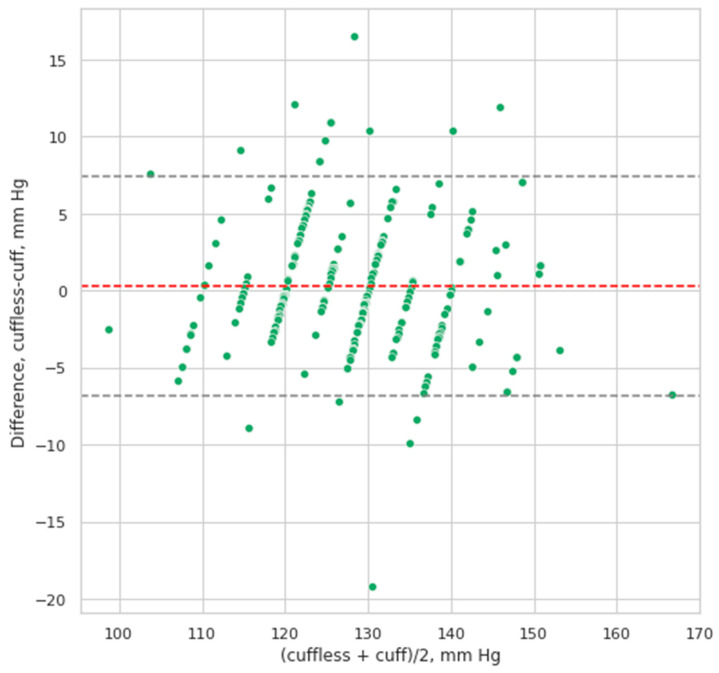
Bland–Altman plot of systolic blood pressure values derived from cuff based versus cuffless measurements. The mean difference between the cuffless–cuff values for DBP was 0.35 ± 2.95 mm Hg, the 95% CI of mean difference was (0.093; 0.61), according to the Student’s *t*-test results, the mean was not equal to 0 (*p* = 0.008). Here the cuffless method also slightly underestimates DBP. The correlation between the 2 measurement techniques for DBP was r = 0.87 (Spearman’s correlation, *p* = 0.002) (Figure 5).

The Bland–Altman analysis demonstrated that the values of SBP and DBP calculated by the cuffless smartphone-case based BP device correlated well with the values measured by using sphygmomanometer with a cuff. The mean difference between the values definite by CardioQVARK for SBP was 0.32 ± 3.63 mm Hg. The 95% confidence interval (95% CI) of mean difference was (0.003; 0.64), according to the Student’s *t*-test results, the mean was not equal to 0 (*p* = 0.048). This means that CardioQVARK slightly overestimates SBP. The correlation (r, Spearman’s correlation) between the two measurement techniques for SBP was 0.89 (*p* = 0.001) (Figure 4).

**Figure 7:**
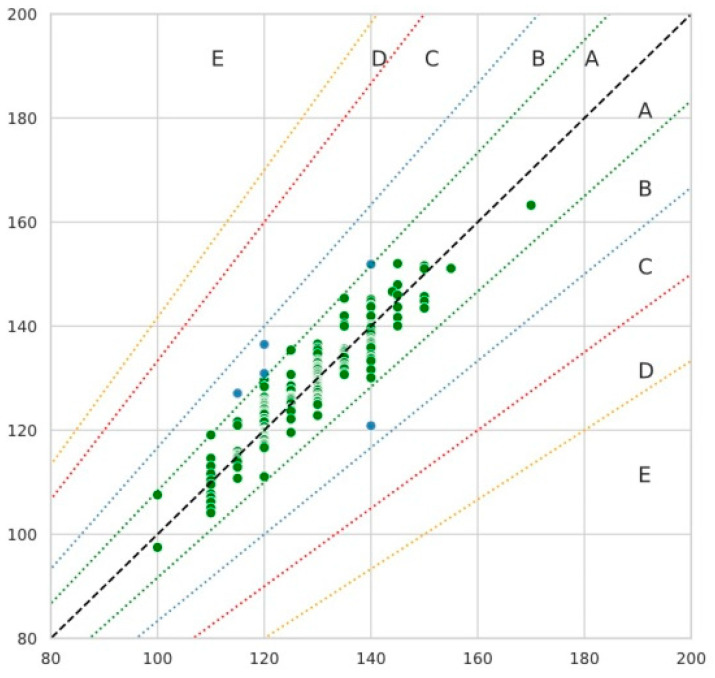
Zonal error grid analysis.

Zonal grid error analysis was performed (Figure 7). Five zones were identified. The method shows what percentage of measurements was found to be clinically accurate.

Column X shows SBP measured by the cuff (mm Hg); Column Y shows SBP measured by the CardioQVARK device. Most of the measurements performed by the device are in zone A.

As an example, if we have cuff value of 140 mm Hg, the zones will be distributed as follows:

- Zone A—predicted value is strictly between 128.3 and 151.7 mm Hg;
- Zone B—predicted value is between 116.7 and 128.3 mm Hg or from 151.7 to 163.3 mm Hg;
- Zone C—predicted value is between 93.3 and 116.7 mm Hg or from 163.3 to 186.7 mm Hg;
- Zone D—predicted value is between 81.7 and 93.3 mm Hg or from 186.7 to 198.3 mm Hg;
- Zone E—predicted value less than 81.7 mm Hg or above 198.3 mm Hg.

The sensitivity and specificity of the cuffless blood pressure measurement method in detecting prehypertension and hypertension were also calculated. The sensitivity of the new method in detecting prehypertension was 97.7% and specificity was 98.2% (accuracy 98%). The sensitivity in detecting hypertension was 95.3% and specificity was 99.8% (accuracy 99.2%). Thus, the cuffless blood pressure measurement method was quite accurate.

## 4. Discussion

Our results of blood pressure measurements by using the smartphone-case based CardioQVARK single-channel ECG monitor with PPG pulse wave recordings are promising. The development of such new technologies for remote patient monitoring without the use of a cuff may help to allow better BP control for many people.

Traditionally, cuff-based devices are used for BP measurements. The measurement of BP using a cuff may be difficult in some patients, which is of particular importance for patients with obesity, where it can be difficult to choose an appropriate cuff size. In addition, sphygmomanometers need to be regularly checked and calibrated (which is widely unknown by patients and even by health professionals). Such inconvenience has contributed to prevent the widespread use of BP monitoring in particular for telemetric applications. The use of cuffless blood pressure measurement has a great potential to simplify and facilitate the BP measurement for many patients. The fact that it is easy to add a special case to a commercially available smartphone is another advantage allowing widespread application. It allows for greater patient autonomy, convenience, and perceived control over management of their chronic condition. The ability to measure BP at any time in most of the places is another advantage. Home-based BP measurements may be more efficacious at lowering systolic and diastolic BP than office-based measurements. Telemetric transmission of the results from home-based measurements may ease interventions such as medication titration. As a result, better BP control translates to decreased morbidity and mortality in patients with hypertension.

The development of non-invasive portable blood pressure monitors has begun many years ago. Currently there are several portable blood pressure monitors [7,8,9,10,13,14,23,29,30,31,32,33,34,35]. They include smartphone applications, wearables, and medical T “tricorders”. However, there are several limitations pertaining to the current available cuffless BP technology: First, the majority of the current devices have not undergone a validation protocol, and as such, their accuracy and precision is not confirmed. Second, the measurements based on ECG, PTT, and tonometry are sensitive to motion artifacts and loss of signal with movement. Third, it is presently unclear for all the currently available cuffless BP technologies how the measured values will relate to clinical or cuff-based home BP measurements in real life. This applies also to our study setting.

There are several machine learning algorithms to determine BP based on PPT [19,20,21,22,23,24,25,26,27,28]. In our study, ECG and PPT were simultaneously analyzed. Registration was carried out using a smartphone case. The algorithm we used in the study simultaneously evaluates ECG and PPT parameters recorded with a smartphone case. Device and application are combined to one unit and were registered with the Federal Service for Surveillance in Healthcare № RZN 2019/8124 on 15 February 2019. The major advantages of our device for cuffless BP determination include: 1. data acquisition is simple by using a smartphone-case based device allowing combined ECG and PPG acquisition; 2. there are no additional devices needed to be connected to the phone; 3. the device can be used without previous knowledge and learning and without the need for assistance by a medical professional.

We found only one study with a comparable number of patients (572) where results from a cuffless method was compared with those from invasive BP measurement (35). In this study, the mean biases for the validation cohort were −2.98 ± 19.35, −3.38 ± 10.35 for SBP, and −3.65 ± 8.69 mm Hg for DBP. Thus, data from this study are not much different from our results. In our study the mean difference between the values definite by CardioQVARK for SBP was 0.32 ± 3.63 mm Hg. The 95% confidence interval (95% CI) of mean difference was (0.003; 0.64), according to the Student’s *t*-test results, the mean was not equal to 0 (*p* = 0.048). The correlation (r, Spearman’s correlation) between cuff and cuffless measurements for SBP was 0.89 (*p* = 0.001). The mean difference between the cuffless–cuff values for DBP was 0.35 ± 2.95 mm Hg, the 95% CI of mean difference was (0.093; 0.61), and, according to the Student’s *t*-test results, the mean was not equal to 0 (*p* = 0.008).

We expected to obtain even more accurate results by measuring blood pressure repeatedly in the same patient with parallel recordings of ECG and PPG and cuff base measurements, and by entering the readings into the CardioQVARK program. Based on the entered data, the algorithm could further be adjusted individually.

### Limitations

In our study, less than 5% of patients had very low (≤100/70 mm Hg) or very high blood pressure (≥160/110 mm Hg). Consequently, the obtained results cannot be extended to the general population of patients with hypertension. Therefore, further studies are needed to better determine the accuracy and reliability of the method for very low and for higher BP values. It is also worth investigating the effect of noise on the estimation accuracy of machine learning models for BP evaluation. This is a rather important point, since the determination of cuffless blood pressure depends on the quality of the recorded PPG. Furthermore, we compared the determination of blood pressure between a non-cuffed method and a non-invasive method, which is a limitation, hence no comparison with the “Gold” standard of invasive blood pressure measurement was made. Finally, the measurements were consecutive and not simultaneous. These limitations require further investigation to ensure that the method is fully applicable.

## 5. Conclusions

First results of cuffless blood pressure measurements using the smartphone-based CardioQVARK single-channel ECG monitor with PPG pulse wave recordings are encouraging. However, further research is needed to improve accuracy and reliability before it can be used in the majority of patients.

## Figures and Tables

**Figure 5:**
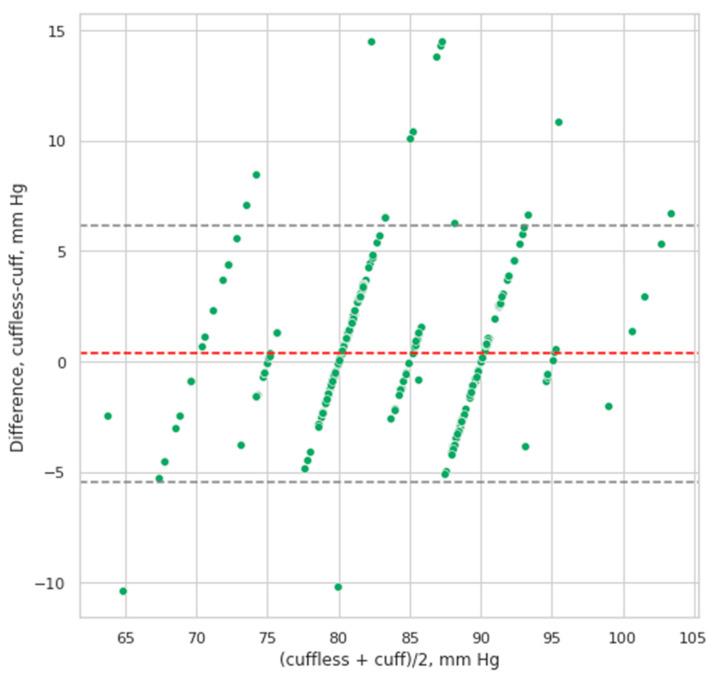
Bland–Altman plot of diastolic blood pressure values derived from cuff based versus cuffless measurements. At different pressure levels in patients, there were no statistically significant differences between the two methods. On average, the pressure varied by 2–4 mm Hg. (Figure 6).

**Figure 6:**
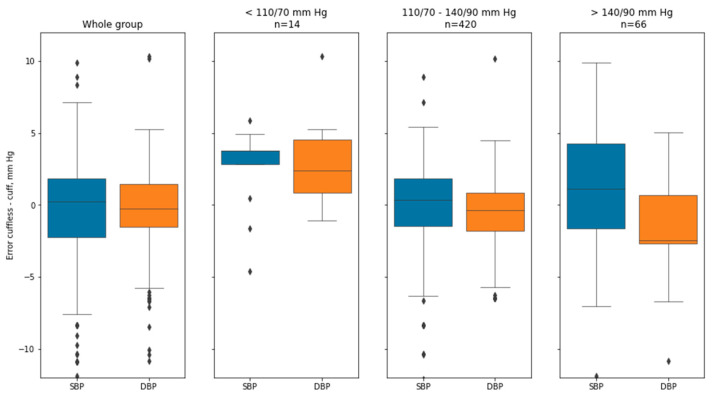
Boxplots of deviations between the two methods in the whole group (*n* = 500) and in three subgroups (BP < 110/70, *n* = 14; 110/70–140/90, *n* = 420; >140/90, *n* = 66) according to the cuff-based results. Mean and median error differed in different subgroups. Wilcoxon rank test was used to access the significance of errors. The cuffless method overestimated BP compared to the cuff-base method significantly in patients with BP values < 110/70 mm Hg both in the SBP and in the DBP subgroup, and underestimated DBP in the subgroups with patients with BP values 110/70–140/90 mm Hg and >140/90 mm Hg. SBP = systolic blood pressure, DBP = diastolic blood pressure (Table 1).

**Table 1:** Mean and median cuffless–cuff errors, mm Hg.

Group and Subgroups According to Cuff Measurements	M ± SD	Me [25%; 75%]	Wilcoxon Test, *p*
++			0.499
Whole group/DBP, mm Hg	−0.35 ± 2.96	−0.24 [−1.55; 1.46]	0.069
<110/70 mm Hg/SBP, mm Hg	2.66 ± 2.94	3.79 [2.82; 3.79]	0.017
<110/70 mm Hg/DBP, mm Hg	2.75 ± 2.93	2.42 [0.86; 4.55]	0.003
110/70–140/90 mm Hg/SBP, mm Hg	−0.17 ± 3.21	0.33 [−1.45; 1.84]	0.159
110/70–140/90 mm Hg/DBP, mm Hg	−0.75 ± 2.45	−0.36 [−1.82; 0.83]	<0.001
>140/90 mm Hg/SBP, mm Hg	0.77 ± 4.49	1.11 [−1.61; 4.29]	0.140
>140/90 mm Hg/DBP, mm Hg	−1.23 ± 3.18	−2.49 [−2.66; 0.70]	0.007

Reference blood pressure values in subgroups were determined based on blood pressure measurements using a cuff-based method. SBP = systolic blood pressure, DBP = diastolic blood pressure.
